# Gut Microbiome of the Critically Endangered New Zealand Parrot, the Kakapo (*Strigops habroptilus*)

**DOI:** 10.1371/journal.pone.0035803

**Published:** 2012-04-18

**Authors:** David W. Waite, Peter Deines, Michael W. Taylor

**Affiliations:** 1 Centre for Microbial Innovation, School of Biological Sciences, The University of Auckland, Auckland, New Zealand; 2 Institute of Natural Sciences, Massey University, Auckland, New Zealand; Argonne National Laboratory, United States of America

## Abstract

The kakapo, a parrot endemic to New Zealand, is currently the focus of intense research and conservation efforts with the aim of boosting its population above the current ‘critically endangered’ status. While virtually nothing is known about the microbiology of the kakapo, given the acknowledged importance of gut-associated microbes in vertebrate nutrition and pathogen defense, it should be of great conservation value to analyze the microbes associated with kakapo. Here we describe the first study of the bacterial communities that reside within the gastrointestinal tract (GIT) of both juvenile and adult kakapo. Samples from along the GIT, taken from the choana (≈throat), crop and faeces, were subjected to 16 S rRNA gene library analysis. Phylogenetic analysis of >1000 16 S rRNA gene clones, derived from six birds, revealed low phylum-level diversity, consisting almost exclusively of *Firmicutes* (including lactic acid bacteria) and *Gammaproteobacteria*. The relative proportions of *Firmicutes* and *Gammaproteobacteria* were highly consistent among individual juveniles, irrespective of sampling location, but differed markedly among adult birds. Diversity at a finer phylogenetic resolution (i.e. operational taxonomic units (OTUs) of 99% sequence identity) was also low in all samples, with only one or two OTUs dominating each sample. These data represent the first analysis of the bacterial communities associated with the kakapo GIT, providing a baseline for further microbiological study, and facilitating conservation efforts for this unique bird.

## Introduction

The kakapo (*Strigops habroptilus*) is one of the world's rarest bird species, with only 126 individuals remaining on two predator-free islands off New Zealand's south coast. Endemic to New Zealand, the kakapo possesses a range of behaviours and physiological characteristics that make it unique: it is the world's heaviest parrot, the only flightless parrot and the only parrot to carry out lek breeding [1]. Due to a combination of infrequent mating, low clutch numbers, and poor defense against mammalian predators the kakapo has been pushed to the verge of extinction [2], [3], though intensive conservation efforts by New Zealand's Department of Conservation have recently reversed the decline in numbers. Research programs into kakapo ecology, nutrition and genetics are well established and a management program has been enacted with the aim of restoring the kakapo population in New Zealand. Such practices as confining birds to predator-free islands, supplementary feeding, breeding programs and constant human supervision of both newborn chicks and adults have had a marked effect on the kakapo population – from just 62 remaining individuals in 1991 [4] to the current level. By contrast, the potentially important roles of symbiotic microorganisms in kakapo nutrition and pathogen defense remain unstudied, although positive bacterial influence on the gastrointestinal tract (GIT) was first observed in vertebrates almost 50 years ago [5].

The interactions between hosts and GIT-associated bacterial communities have been the subject of intense study in mammals, particularly humans [6], [7], with murine models often used to demonstrate causal links between microbes and aspects of host health [8]. Among avians, microbial research has mainly focused on either pathogen detection, or effects on weight gain in broiler chickens [9]. In the last decade, the study of microbial communities in the GIT of birds has become commonplace, with cultivation-dependent and -independent methods used to examine microbial presence and activity within avian gastrointestinal environments. The microbial communities associated with commercially farmed species such as turkey [10] and ostrich [11] have been investigated, as well as a range of wild birds, including parrots [12], [13] and the South American hoatzin [14], [15], [16], and their roles in bird fitness extend far beyond involvement in digestion and nutrient uptake. For example, studies on the effect of feather-degrading bacteria on mate selection and breeding fitness have revealed novel mechanisms through which bacteria can influence the lifecycle of their host [17], [18].

Links between microbial community structure and increased energy harvest from food have been demonstrated for a wide range of organisms by a variety of indirect techniques [19], [20], [21], [22]. In controlled murine models, these effects can be shown at a much more direct level, with gnotobiotic (germ-free) rodents used as controls in experiments that demonstrate the role of bacteria in regulating gene pathways in a range of organs [23], [24], [25], [26]. Microbes isolated from a particular host gut have been shown to be highly adapted to the host environment with the community being shaped by host-specific factors in a range of organisms [27], [28], [29]. Microbes transplanted into a new gnotobiotic host provide significantly reduced benefits to the new host [25]. Stimulation of the host immune system by GIT microbes has also been recognized in response to both viral and bacterial challenge [30], [31], [32], and development of gut-associated lymphoid tissues is increased in conventionally raised mice compared to their germ-free counterparts [33], [34].

With such varied and important roles being influenced by microbes, the lack of an accurate baseline description of kakapo-associated microbes represents a major gap in our knowledge of kakapo biology. Identification of the indigenous microbial community would be of great value to conservation efforts by enabling identification of allochthonous – potentially pathogenic – microbes. The existing literature surrounding kakapo-associated bacteria has so far focused on detecting and responding to pathogen outbreaks. Such an event occurred in 2004, when three kakapo died from erysipelas within 72 hours of translocation. The birds had been checked for known pathogens [35], and erysipelas had not previously been observed in kakapo [36]. While attacks from previously unidentified pathogens are unavoidable, this highlights an area in which molecular microbiology could play a key role in aiding kakapo recovery efforts, through the use of specific, high-sensitivity molecular probing techniques to detect pathogens before their numbers expand to levels that affect the bird.

Human interaction with wild birds can influence the composition of the GIT community [13], [37], and the potential for human impact on the kakapo GIT community is great (although unavoidable). In times of sickness, wild kakapo are taken into captivity and frequently treated with broad-spectrum antibiotics to combat pathogens. In captivity kakapo are fed a diet supplemented with fruit and pellets not available in the wild and hand-reared chicks are fed on bird formula exclusively until approximately 30 days of age [38]. A better understanding of kakapo microbiology carries clear potential for aiding conservation of this endangered bird, yet there are also sound academic reasons for researching this area. The kakapo diet consists mainly of shoots and leaves, and there has been speculation that kakapo may utilize microbes in the foregut to ferment ingested plant material [39]. While this process is common in ruminants (e.g. cattle and sheep) it is almost unknown among avians, with only the hoatzin known to use the foregut to facilitate fermentation [40]. The hoatzin, sole member of the family *Opisthocomidae*, exploits a diverse microbial community in its enlarged crop to aid in digestion, utilising up to 40 bacterial phyla as well as archaea to ferment plant material in the crop [15]. The kakapo has been suggested as a possible candidate for foregut fermentation due to its lack of a cecum, which is the primary site of hindgut fermentation [41], and its similar diet to the hoatzin.

The key aim of this study was to document the microbial community of the kakapo digestive tract in both newly hatched chicks and adults, using samples derived from both the fore- and hindgut to ensure maximum coverage of the GIT. 16 S rRNA gene analysis was used to identify bacteria at each sampling site, and the samples were compared to test for changes in community structure along the GIT. This study represents the first step in a wider investigation of the kakapo microbiome, with the ultimate goal of aiding conservation and management of this critically endangered bird.

## Results

### Bacterial community composition within the kakapo gastrointestinal tract

**Figure 1:**
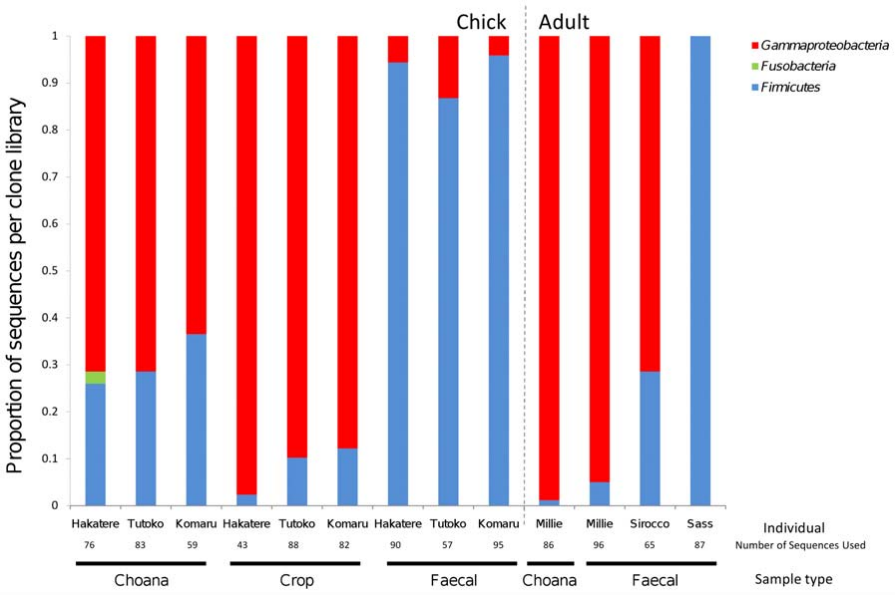
Phylum-level distribution of kakapo-derived 16 S rRNA gene sequences. Phylum-level affiliation of 16 S rRNA gene sequences obtained from the kakapo GIT. Samples to the left of the dotted line represent clone libraries derived from juveniles, and samples on the right represent adult-derived sequences.

**Figure 2:**
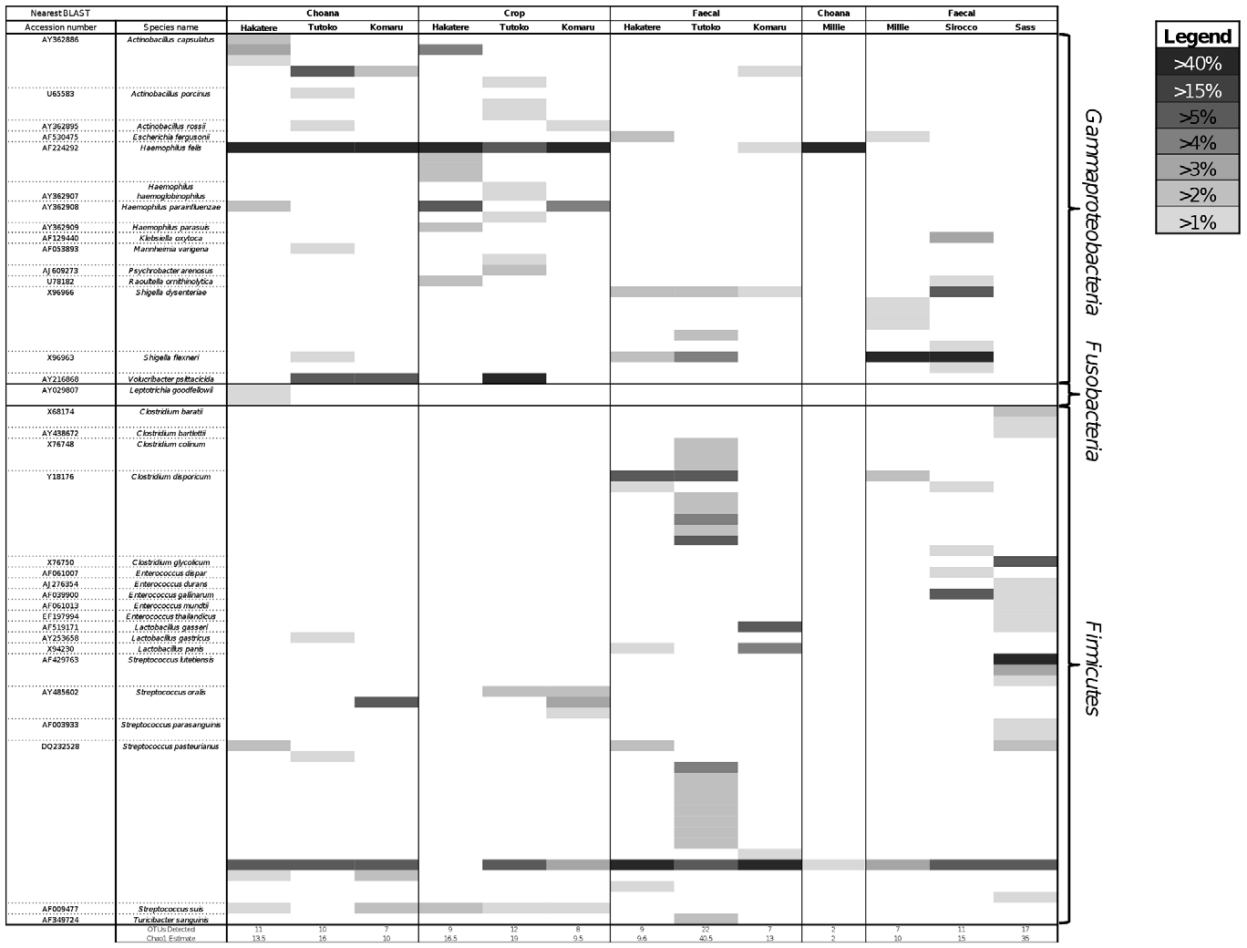
OTU-level distribution of kakapo-derived 16 S rRNA gene sequences. OTU-level affiliation of 16 S rRNA gene sequences obtained from the kakapo GIT. Values in this heatmap are scaled as a proportion of the total number of sequences per clone library. Observed numbers of OTUs at 99% sequence similarity are provided below the figure, as well as the estimated diversity for each library using the Chao1 estimator.

**Figure 3:**
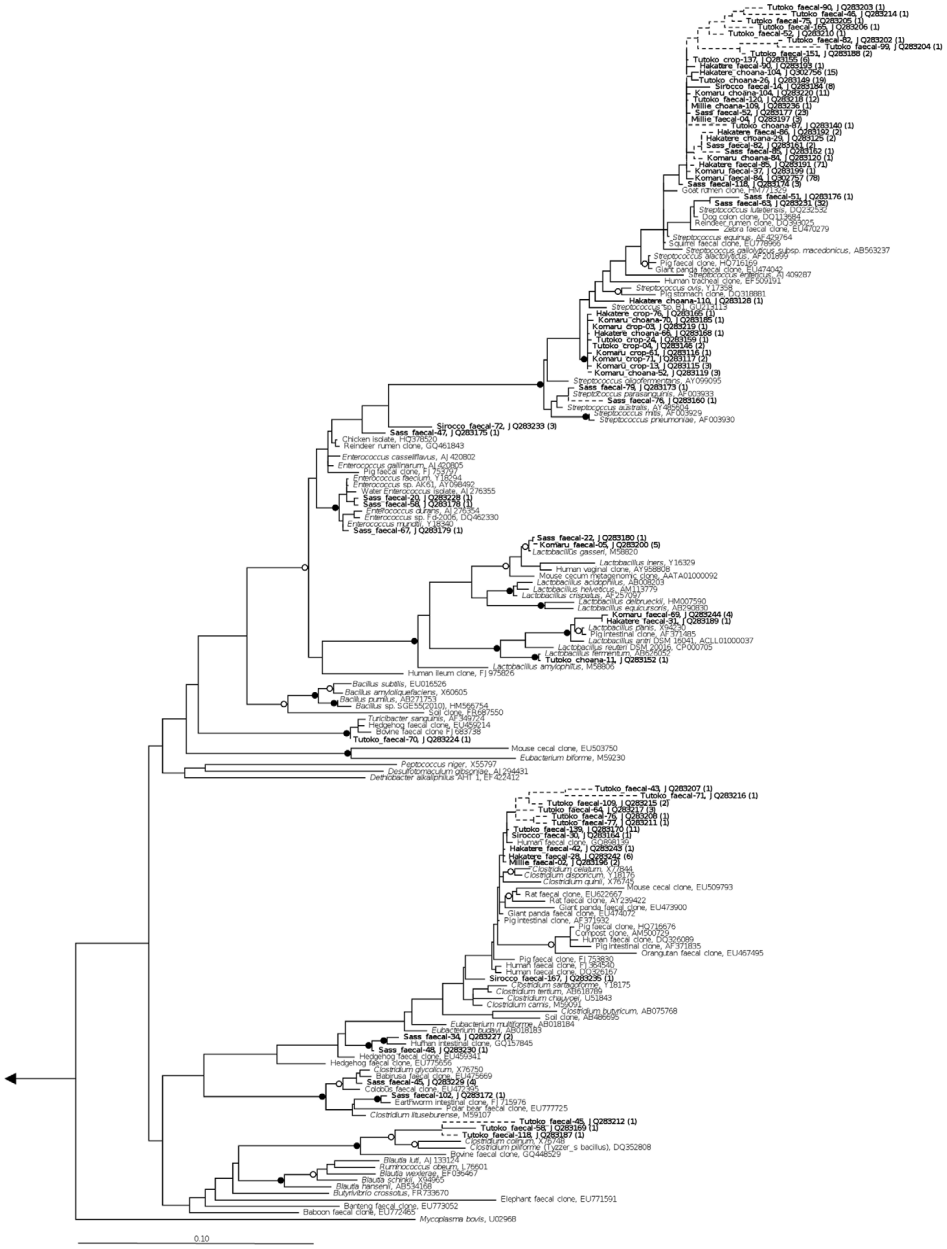
Phylogeny of *Firmicutes*-associated 16 S rRNA gene sequences derived from kakapo. 16 S rRNA gene-based phylogenetic analysis of *Firmicutes* recovered from kakapo samples. Solid junctions represent >90% bootstrap support, and hollow junctions >75%. Kakapo-derived sequences are in bold. Dashed lines indicate sequence length <1200 bp. Branch lengths were calculated using the maximum-likelihood method RAxML, using sequences >1200 bp in length, and short sequences were added subsequently using the Parsimony Interactive tool in ARB. Bootstrap values were calculated using maximum parsimony with 5000 resamplings. Scale bar, 10% sequence divergence.

**Figure 4:**
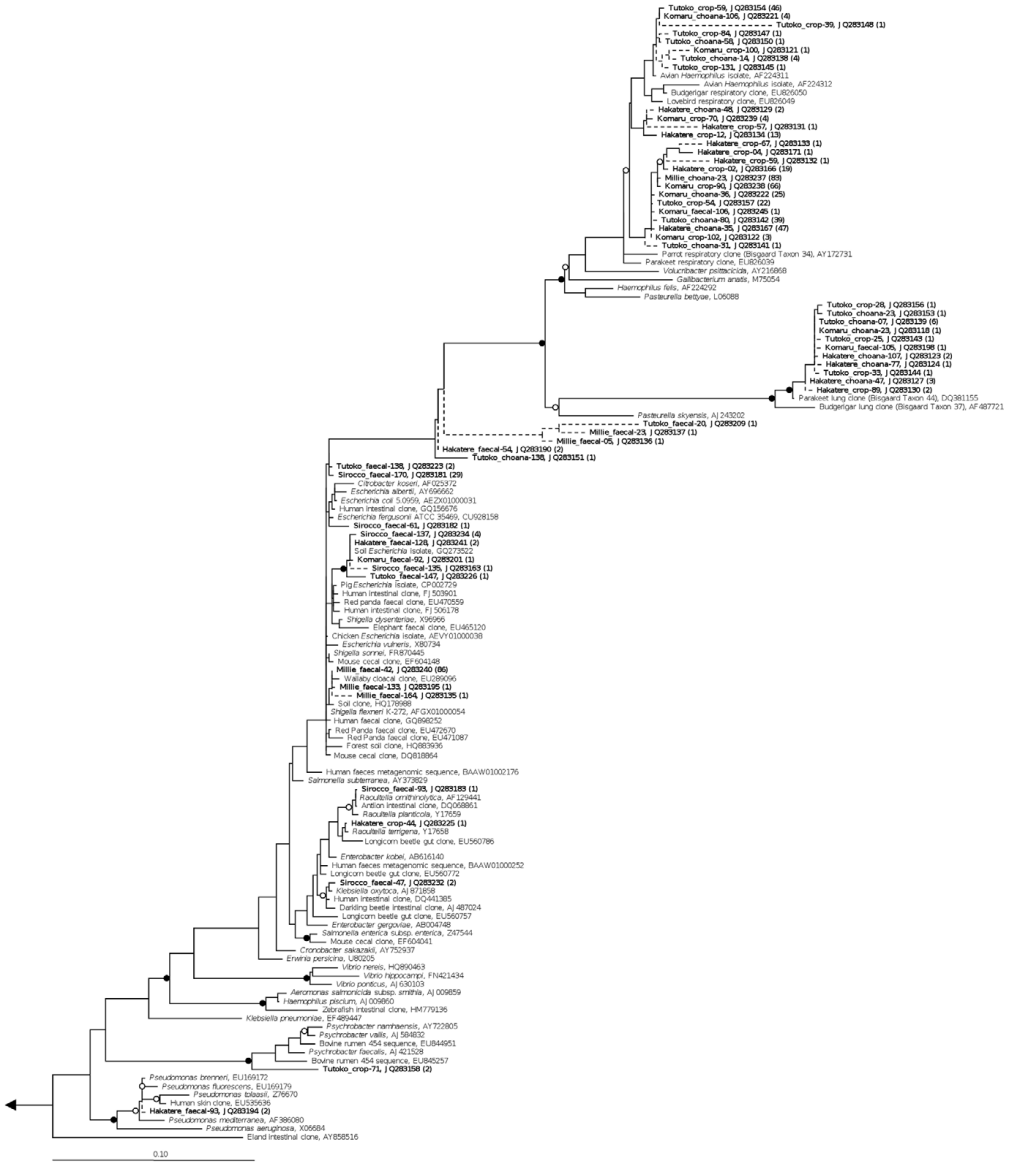
Phylogeny of *Gammaproteobacteria*-associated 16 S rRNA gene sequences derived from kakapo. 16 S rRNA gene-based phylogenetic analysis of *Gammaproteobacteria* found within kakapo samples. Solid junctions represent >90% bootstrap support, and hollow junctions >75%. Kakapo-derived sequences are bolded. Dashed lines indicate sequence length <1200 bp. Branch lengths were calculated using the maximum-likelihood method RAxML, using sequences >1200 bp in length, and short sequences were added subsequently using the Parsimony Interactive tool in ARB. Bootstrap values were calculated using maximum parsimony with 5000 resamplings. Scale bar, 10% sequence divergence.

Bacterial 161S rRNA gene amplification was successful for all samples, whereas no archaea were amplified from any samples. A total of 1007 clones yielded high-quality sequence that passed chimera checking. The phylum *Firmicutes* was present in all libraries, and *Gammaproteobacteria* present in all except one (Sass). Slight representation from *Fusobacteria* was seen in a single chick choana sample (Fig. 1). When sequence data were dereplicated into 99% OTUs it was revealed that most of the sequences belonged to only a few key OTUs, such as *Haemophilus felis* and *Streptococcus pasteurianus* (Fig. 2). A Chao1 diversity estimator for each clone library was calculated at the 99% OTU level, and in almost all cases the expected number of OTUs per library was close to the observed number. The remainder of the diversity in each library was split among several low-abundance OTUs. Phylogenetic trees of kakapo-associated *Firmicutes* and *Gammaproteobacteria* are shown in Figs. 3 and 4, respectively.

The extent of differences in bacterial community composition between samples was tested at the OTU level using an unweighted UniFrac analysis. Sequences obtained from kakapo chick, and adult faecal, samples were pooled according to sample type. Sequence data obtained from the single adult choana swab were not included in statistical comparison. Pairwise comparisons were made between sample types to test the null hypothesis that the bacterial community is homogeneous throughout the GIT. Significant differences in community structure were observed between the chick choana/crop (p<0.001) and crop/faeces (p = 0.002), but not between the choana/faeces. Between chick and adult faecal samples, no significant difference was seen. Given that Sass and Sirocco had been subject to considerable human intervention prior to sampling, their faecal samples were compared separately to the chick samples and wild adult sample (Millie). The faecal communities of Sass/Sirocco were significantly different from those of the wild adult (p = 0.002), but did not differ significantly from the chick samples. There was no significant difference between the wild adult and chick faecal samples.

### Determination of *Bacteroidetes* and *Archaea* sensitivity

*Bacteroidetes* are common GIT-associated bacteria in many vertebrates, but were not detected in any of the kakapo samples. As certain DNA extraction techniques can lead to under-representation of *Bacteroidetes* in a sample [42], we tested whether our DNA extraction methods are able to detect the presence of *Bacteroidetes* within faecal and swab samples. *Bacteroidetes* DNA was successfully detected in all spiked faecal and swab samples (data not shown), down to approximately 0.15% of bacterial cell load, indicating that *Bacteroidetes* were not excluded by our DNA extraction protocols. Similarly, while *Archaea* were not detected in un-spiked kakapo faeces, the archaeal 16 S rRNA gene could be detected when swab and faecal samples were spiked with archaeal cells, down to approximately 0.4% of cell load.

## Discussion

This paper describes the first molecular examination of the bacterial communities within the kakapo GIT, and provides evidence that qualitative differences exist between sites sampled throughout the GIT. The kakapo GIT appears to harbor a low-diversity community of microbes, with essentially only two phyla detected, *Gammaproteobacteria* and *Firmicutes*. Microbes in the kakapo GIT are abundant, with both cultivation-based measurements and DAPI cell counts indicating a microbial cell density in the order of 10^10^ cells per gram of faecal material (data not shown), yet each sample is dominated by only a few genera, typically *Haemophilus*, *Streptococcus*, and *Clostridium*. As the *Fusobacteria* discovered were only in a single sample, and found in low abundance, it is possible that their presence represents some form of contamination which occurred during sampling or DNA extraction. The *Fusobacteria*-associated sequences were similar to isolates and clones of the genus *Leptotrichia*, a bacterium commonly found in the human oral cavity [43].

At the phylum level, bacterial diversity is well conserved among all chicks sampled, but within the adults there is large variation in terms of relative abundance of each phylum. This may be explained by a range of factors regarding the adults, such as the frequent handling of Sirocco and, to a lesser extent, Sass, or the age difference between Sass and Millie/Sirocco. The bird Sass died several weeks after the collection of faecal samples, but not due to pathogen-related illness, and had not been treated with antibiotics prior to sampling (which can disrupt the GIT community [44], [45]). Subject age has been linked to a shift in the bacterial gut community in humans [46], [47] and mice [48], so it is conceivable that such a community change may be a natural phenomenon. While functional roles of the bacteria detected in this study can only be speculated upon, those bacteria encountered in the kakapo GIT correspond to genera commonly observed in other herbivores. In a study of the gut microbiota of deer it was recognized that *Streptococcus* played a role in degrading tannins ingested by the host animal, and many of the *Streptococcus* detected in the kakapo clone libraries were closely related to this species (Fig. 3, *Streptococcus gallolyticus* sub. *macedonicus*, AB563237) [49]. Most of the bacterial genera detected throughout the kakapo GIT are known anaerobic fermenters, capable of converting sugars such as glucose and cellulose into acids such as acetate, which are utilized by the host. Members of the genus *Clostridium* are frequently identified as cellulolytic [50], [51], [52], [53], and have been found to increase in proportion within the herbivore gut in the absence of starch [54]. The inability to detect *Bacteroidetes* in a parrot, using either 16 S rRNA-based techniques or cultivation, is not unique to our study [12], [13]. In addition to playing roles in butyrate production and bile acid metabolism [55], [56], *Bacteroidetes* are well-characterized degraders of starch and cellulose in the gut [57], [58], [59], [60]. Historically the kakapo have relied on a low-starch diet [61], which may have selected against *Bacteroidetes* colonization, as diet has been identified as one of the factors that shape gut microbiota [62], [63], [64], [65]. While the DNA extraction method utilized in this study is capable of extracting detectable levels of DNA from *Bacteroidetes* comprising less than 1% of the community, it is conceivable that the inability to detect *Bacteroidetes* stems from low sequence counts compared to those obtained using next-generation sequencing technologies.

Given the endangered status of the kakapo, destructive sampling (via dissection) is not possible. As such, our analyses were limited to swab and faecal samples rather than direct tissue and gut content samples. Although surface swabs may not give a perfect representation of the local bacterial community, they have been previously applied in a range of avian study systems [13], [17], [66], [67], [68] where dissection of the target organism was not feasible. There still exists the potential that mucosa-associated bacteria of the crop may not be recovered through the swabbing of live animals, indicating a potential blind spot in sampling. Nevertheless, it appears that swabbing of the kakapo crop is adequate for differentiating between microbial communities of the choana and crop, despite the fact that any probe into the crop risks potential contamination as the swab passes the choana. The use of faecal samples as a proxy for hindgut bacterial communities has been used extensively in a range of vertebrates, including humans and birds. While several studies have highlighted differences between bacterial recovery from mucosal biopsies and faecal samples [7], [69], this appears to be due to faecal samples containing not only mucosa-associated bacteria that have been shed into the faeces, but also bacteria that colonize the faecal substrate directly. Community data taken from faecal samples contains a reasonable representation of microbes within the hindgut, and differences in faecal microbiota (both at presence/absence and functional levels) have been shown to reflect differences in the intestinal tract of the host [48], [70], although it must be stressed that they do not provide an exact representation of microbial community structure and function within the intestine itself. Based on unweighted UniFrac analysis it appears that the faecal bacterial communities of adults and chick kakapo are not significantly different, which may indicate a vector for inoculation of kakapo chicks with their parents' microbiota. The lack of significant difference between choana and faecal communities in the chicks is not surprising considering the lifestyle of unfledged kakapo chicks. Essentially immobile, the chicks are unable to distance themselves from their own faeces. The chicks studied in this project have since been fledged and given the low population of kakapo and constant attention to the birds, these present an excellent opportunity for longitudinal studies throughout the lifespan of the birds.

Given the low-energy diet of the kakapo and its lack of cecum, it has been speculated that the kakapo may utilize microbially-mediated foregut fermentation to derive additional energy from its food. While this study was not targeted at confirming or rejecting the notion of kakapo foregut fermentation, the possibility that key microorganisms may be resident in the crop rather than hindgut was taken into consideration when planning this study. There are several frequently found bacterial phyla in the microbial community of foregut-fermenting mammals and the hoatzin, predominantly *Firmicutes* and *Bacteroidetes*, with representation from *Verrucomicrobia*, *Actinobacteria* and *Spirochaetes* commonly observed [15], [71]. Methanogenic archaea are also commonly found in the rumen or crop of foregut fermenters [14], [72], [73], [74], [75], [76]. With the exception of *Firmicutes*, none of the above-mentioned taxa were detected in the kakapo samples. In the hoatzin it has been shown that the microbial community of the foregut is similar to that of ruminants [16], but given the apparent absence of so many bacterial phyla in the kakapo crop it is unlikely that the kakapo shares this community structure and gut adaptation. Foregut fermentation is an adaptation to a diet rich in celluloses that the host cannot digest [77], but kakapo do not retain and digest cellulose in the manner seen in ruminants and the hoatzin, typically spitting away masticated plant material after extracting juices from the flesh [61], [78]. Although merely speculative at this stage, it thus seems unlikely that kakapo perform foregut fermentation in the traditional manner.

One observation from this study that may prove to be of future concern is the high number of *Pasteurellaceae*-like sequences within the choana and crop swabs. Many of the sequences were clustered with bacterial genera such as *Haemophilus*, or with several non-cultivated clades commonly detected in the avian respiratory tract, which are frequently found as respiratory pathogens in vertebrates [79]. It has been noted that certain *Pasteurellaceae* which were present in our libraries (Bisgaard Taxon 34, Bisgaard Taxon 44, Fig. 4) are frequently associated with respiratory disease in psittacine birds. Although not all bacterial species in these clades are causative agents of disease, their presence should be considered a warning, as they are often found in sick birds [80]. During the 2011 breeding season several chicks were removed from the nest due to respiratory problems, although this did not cause long-term health issues in the birds (D. Eason, personal communication). While there is no data to imply a causal link between the observed *Pasteurellaceae* and the illness, pathogens do appear to have been introduced to the kakapo population previously through avian vectors [36].

In summary, we performed the first 16 S rRNA-based microbial analysis of the bacteria that inhabit the kakapo GIT. We have shown that the GIT is inhabited by a few key organisms, and that the community composition changes throughout the GIT. Our results also provided preliminary evidence that the human influence on kakapo lifestyle appears to cause a shift in these bacterial communities, although whether this has a positive, negative, or neutral effect on the bird remains unknown.

## Materials and Methods

### Sampling

Samples were obtained from four kakapo on Codfish Island (46°47′S 167°38′E), off the coast of Stewart Island, New Zealand, during the nesting season, between 17^th^ and 23^rd^ April 2011. Two additional faecal samples had previously been collected from adult birds when they were brought to Auckland Zoo. A total of 13 samples were used in this analysis, collected from three unfledged chicks, and three adults. Samples of the upper GIT were taken by Department of Conservation staff using sterile rayon-tipped swabs (Copan, #170KS01), and samples were taken from the choana and crop of chicks, and choana of one adult. The choana is an opening in the roof of the mouth that joins to the nasal passage. Due to difficulties in restraining adults, crop samples could not be taken from adult birds. A faecal sample was collected from all six birds at the time swabbing was performed. As interference with nesting mothers can cause them to abandon the nest, samples from chick parents could not be obtained. Swabs were stored in sterile polypropylene tubes and kept on ice until they were frozen at the ranger hut on Codfish Island. Samples were shipped to The University of Auckland on dry ice, and stored at −20°C upon arrival.

### DNA Extraction

Despite considerable efforts to standardize the DNA extraction procedure, it was necessary to use a different approach for extracting DNA from swab vs faecal samples due to unreliable DNA retrieval from hard-to-obtain swab samples and unwillingness to risk valuable samples. Genomic DNA was extracted from swabs using heat lysis. Swab tips were placed in a 1.5 mL cryotube containing 1 mL extraction buffer (20 mM EDTA, 0.1 M Tris (pH 8.0), 1% CTAB, 56 mM NaCl), briefly vortexed, then incubated at 94°C for 15 min in order to lyse both Gram-negative and Gram-positive cells [81], [82]. The solution was cooled briefly on ice, 300 µL of chloroform/isoamyl alcohol (24∶1 ratio) was added and the solution mixed by inversion, then centrifuged at 13,000 rpm for 5 min at room temperature. The supernatant was transferred to a 2 mL microcentrifuge tube, to which 0.6 volume (vol) isopropanol and 0.1 vol 3 M sodium acetate (pH 5.2) were added. Samples were incubated overnight at −20°C then centrifuged at 13,000 rpm at 4°C for 30 min. The supernatant was discarded and the pellet washed twice with ice-cold 70% ethanol followed by centrifugation at 13,000 rpm at 4°C for 10 min. Samples were dried and suspended in 50 µL UltraPure water (Invitrogen).

Genomic DNA was extracted from kakapo faecal samples using a variation on an extraction protocol previously described [83]. 100 mg of faeces were suspended in 1 mL of 70% ethanol with 200 mg of 0.1 mm zirconia/silica beads in a 1.5 mL cryotube. Samples were agitated using a FastPrep FP120 bead beater, at 5.5 ms^−1^ for 30 s, followed by centrifugation at 13,000 rpm for 5 min and removal of supernatant. 1 mL of extraction buffer was added to each tube in addition to 30 mg of polyvinylpolypyrrolidone (PVPP), before being agitated using the previous settings. Samples were then incubated at 65°C for 30 min, with gentle mixing every 10 min. Samples were centrifuged at 13,000 rpm for 5 min and the supernatant was transferred to a fresh 1.5 mL microcentrifuge tube containing 0.5 mL of chloroform/isoamyl alcohol solution (24∶1 ratio) and inverted to mix. Samples were centrifuged at 13,000 rpm for 5 min, before the supernatant (approx. 1 mL) was transferred to a 2 mL microcentrifuge tube containing 0.6 vol isopropanol and 0.1 vol 3 M sodium acetate (pH 5.2). Samples were mixed then incubated on ice for 1 h, then centrifuged at 13,000 rpm at 4°C for 1 min to remove any remaining sediment (presumed to be leftover SDS). The supernatant was transferred to a new microcentrifuge tube and centrifuged under the same conditions for 30 min. The supernatant was removed and the pellet washed twice using ice-cold 70% ethanol followed by 10 min centrifugation at 13,000 rpm, 4°C. The pellet was dried and resuspended in 50 µL UltraPure water (Invitrogen).

### PCR and clone library construction

PCR was performed using the forward primer 616V and reverse primer 1492R, targeting *Escherichia coli* positions 8–27 and 1492–1513 respectively, to amplify bacterial 16 S rRNA genes, and 21F/958R for archaeal 16 S rRNA genes (Table 1). Reactions were conducted in 25 µL volumes, containing 20 mM Tris-HCl, 50 mM KCl (buffer), 1.5 mM MgCl_2_, 25 µM of each dNTP, 2.5 µM of each primer, 0.5 units Taq polymerase and 1.0 µL of extracted DNA template. Cycling conditions for the 616V/1492R primer pair were as follows: initial denaturing at 94°C for 5 min, 30 cycles of 94°C for 45 s, 57°C for 45 s and 72°C for 1.5 min, followed by a final elongation step at 72°C for 7 min. Cycling conditions for 21F/958R were described previously [84]. In order to successfully amplify from faecal samples, the addition of 2% bovine serum albumin per tube was required [85]. Cloning was performed using the P-GemT Easy Vector kit (Promega, Inc, Madison WI, USA) following the manufacturer's instructions. Approximately 96 clones from each of the 13 clone libraries were selected for sequencing (Macrogen Inc, Seoul, South Korea).

**Table 1:** Sequences for primers used in this study.

Primer Name	Sequence	Reference
616V	5′-AGAGTTTGATYMTGGCTCAG-3′	[91]
1492R	5′-GGTTACCTTGTTACGACTT-3′	[92]
21F	5′-TTCCGGTTGATCCYGCCGGA-3′	[93]
958R	5′-TCCGGCGTTGAMTCCAAT T-3′	[93]
341-GC	5′-[30×G/C]-CCTACGGGAGGCAGCAG-3′	[94]
518R	5′-ATTACCGCGGCTGCTGG-3′	[95]

### Phylogenetic Analysis

Sequences were analyzed using the Geneious software package [86] and low-quality data from the ends of each sequence removed. Chimeras were identified with the Pintail algorithm using the Mallard software package [87] and subsequently removed from the data set. Sequences were aligned via the SINA web aligner [88] and imported into ARB using the SILVA 108 database [89]. Sequence data were divided into operational taxonomic units (OTU) of 99% sequence identity using mothur [90] and one sequence to represent each OTU per sample was used in tree construction. Sequences representing each OTU were submitted to the DDBJ/EMBL/GenBank databases under accession numbers JQ283115–Q283245, JQ302756, and JQ302757. Phylogenetic trees were constructed in ARB using the maximum likelihood method RAxML. Bootstrap values were calculated using 5000 parsimony replications. Unweighted UniFrac analyses were performed in mothur to statistically compare bacterial community composition among different sample types.

### Determination of *Bacteroidetes* and *Archaea* sensitivity

A pure culture of *Chryseobacterium formosense* (phylum *Bacteroidetes*), originally isolated from wastewater, was obtained from a colleague and cultivated at the original isolation conditions (R2A broth, 28°C for 48 h, C. Brown, personal communication). *C. formosense* cells were added to samples of kakapo chick faeces at proportions down to 0.15% total bacterial cell load, calculated through enumeration of *C. formosense* via plating counts, and DAPI staining of faecal samples, then subjected to both extraction methods detailed previously. A fragment of the 16 S rRNA gene was amplified using the 341-GC/518R primer pair. Cycling conditions consisted of an initial denaturing step at 94°C for 5 min, followed by 25 cycles at 94°C for 1 min, 60°C for 1 min, and 72°C for 30 s, then a final elongation step at 72°C for 5 min. The product was analyzed using denaturing gradient gel electrophoresis (DGGE) with a denaturing gradient of 40–70%. A positive control of pure *C. formosense* DNA was used as an indicator of a *Bacteroidetes* band in the gel pattern. A pure culture of *Methanosarcina acetivorans* (domain *Archaea*, strain DS2834) was obtained from the German Collection of Microorganisms and Cell Cultures (DSMZ) and added to samples of kakapo chick faeces at proportions down to 0.4% total cell load. A fragment of the archaeal 16 S rRNA gene was amplified using the 21F/958R primer pair. Cycling conditions were described previously [84]. Samples were visualised on a 1% agarose gel and analysed using the BioRad Gel Doc imaging system.
